# Remote energy sources for mixing in the Indonesian Seas

**DOI:** 10.1038/s41467-022-34046-6

**Published:** 2022-11-01

**Authors:** Chengyuan Pang, Maxim Nikurashin, Beatriz Pena-Molino, Bernadette M. Sloyan

**Affiliations:** 1 Institute for Marine and Antarctic Studies, University of Tasmania, Hobart, TAS Australia; 2 ARC Centre of Excellence for Climate Extremes, University of New South Wales, Sydney, NSW Australia; 3 Australian Antarctic Program Partnership, University of Tasmania, Hobart, TAS Australia; 4 Center for Southern Hemisphere Ocean Research, Hobart, TAS Australia; 5 CSIRO Oceans and Atmosphere, Hobart, TAS Australia

**Keywords:** Physical oceanography, Climate and Earth system modelling

## Abstract

The role of the Indonesian Seas in climate is attributed to the intense mixing observed throughout the region. Mixing cools the surface temperature and hence modifies the atmospheric convection centered over the region. Mixing also controls the heat exchange between the Pacific and Indian Oceans by transforming water-mass properties while they transit through the region. Mixing in the Indonesian Seas has long been identified to be driven locally by tides. Here we show that the observed mixing can also be powered by the remotely generated planetary waves and eddies. We use a regional ocean model to show that the Indonesian Seas are a sink of the energy generated in the Indian and Pacific Oceans. We estimate that 1.7 GW of the remotely generated energy enters the region across all straits. The energy flux is surface intensified and characterized by a convergence, implying dissipation and mixing, within the straits and along topography. Locally, energy convergence associated with this process is comparable in magnitude to tidal energy dissipation, which dominates the deep ocean.

## Abstract (web summary)

In addition to locally generated tidal mixing, remotely generated planetary (Kelvin and Rossby) waves and eddies are found to supply energy into the Indonesian Seas, sufficient to drive mixing in the upper ocean at rates inferred from observations.

## Introduction

The Indonesian Seas play a pivotal role in the global ocean circulation and climate by providing the exchange between the Pacific and Indian Oceans and coupling between the ocean and the atmosphere^1,2^. The Indonesian Throughflow (ITF), a system of ocean currents flowing from the Pacific to the Indian Ocean, and the associated transformation of water masses as they circulate through the region are crucial components of the global thermohaline circulation^3^. Heat and freshwater fluxes associated with the ITF have been shown to contribute to the mean state of the Pacific and Indian Oceans^4–9^. Sea surface temperature (SST) within the Indonesian Seas controls air-sea interaction and hence the tropical deep convection and the monsoonal response in this region^10–13^. SST over the Indonesian Seas has also been found to both respond to and modulate the El Niño Southern Oscillation (ENSO) and the Indian Ocean Dipole (IOD) modes of climate variability, which drive precipitation and ocean circulation variability within the entire Indo-Pacific region^14,15^ and globally^16^. Both the SST within the Indonesian Seas^12^ and the inter-basin exchange by the ITF^17^ are sensitive to mixing in the upper ocean.

The Indonesian Seas are host to some of the most intense mixing in the ocean. Observations of the turbulent energy dissipation from the Indonesian Mixing program (INDOMIX)^18^ revealed turbulent energy dissipation rates in the range of 10^−10^–10^−5^ W kg^−1^, corresponding to vertical diffusivity values of 10^−5^–10^−2^ m^2^ s^−1^, orders of magnitude larger than ocean background values of 10^−10^ W kg^−1^ for energy dissipation and 10^−5^ m^2^ s^−1^ for diffusivity^19^. These high turbulence and mixing rates were found in the thermocline and at the base of the mixed layer. More recently, mixing rates inferred from historical conductivity, temperature, and depth (CTD) measurements^20^ and direct microstructure measurements in the Indonesian Seas^21^ indicate that the observed turbulent mixing is localized in a few narrow straits with full-depth integrated dissipation rates of 10–100 mW m^−2^ (1 mW m^−2^ = 10^−3^ W m^−2^). The vertical distribution of high dissipation rates varies across the region. Stations following the western route of the ITF (via Sulawesi Sea, Makassar, Lombok, and Sape Straits) are characterized by one to two orders of magnitude enhancement of the turbulent energy dissipation in the upper 100–200 m compared to deeper energy dissipation rates. Stations following the eastern route of the ITF (via Maluku and Halmahera Seas) show a more uniform, or bottom-intensified, distribution of turbulent energy dissipation with depth.

Breaking of internal tides—internal waves generated by tidal currents interacting with steep topography —is thought to be the dominant driver for the observed mixing in the Indonesian Seas^1,11,22,23^. Recent modeling studies of the internal tide generation in the Indonesian Seas find the strongest generation sites are at the eastern passages of Halmahera, Lifamatola, Sula Straits, and the Sangihe Island chain^22,24^ with the total energy conversion into internal tides within the Indonesian Seas of up to 80-90 GW. The semi-enclosed nature of the Indonesian Seas prevents the radiation of the locally generated tidal energy to other regions, suggesting that most of the internal tide energy is available for mixing within the region. Although the complex internal tide propagation and dissipation processes remain poorly understood^25,26^, the bulk of the internal tide energy is expected to dissipate within the eastern passages and in the deep ocean, where internal tides are primarily generated. Compared against observations, internal tide models also show a bias toward lower dissipation rates in the thermocline^20,22^, implying a potential contribution to the upper ocean mixing from other sources. Previous studies show that Kelvin waves, Rossby waves, and eddies generated remotely by winds in the Indian and Pacific oceans propagate into the Indonesian Seas and drive strong variability in this region^27^. These motions are known to be a significant source of energy in the western boundaries of mid-latitude basins^28^. However, their contribution to mixing in the Indonesian Seas has been overlooked.

**Fig. 1:**
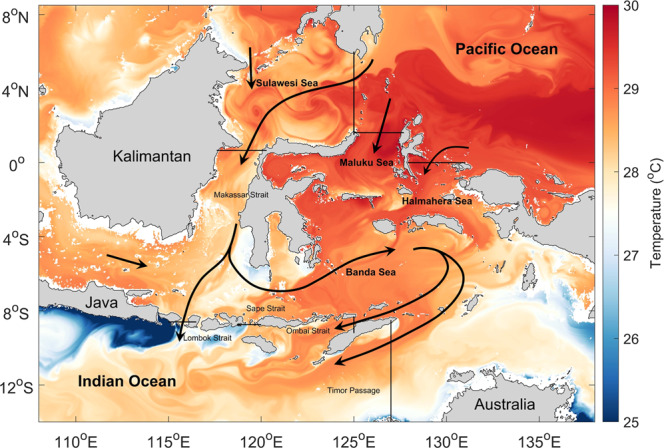
A snapshot of the sea surface temperature in (°C) at 25 m depth at the end of the simulation. Sections across the major entrance and exit straits and passages on the Indian and Pacific Ocean sides of the region used for the total energy flux calculation in Table 1 are shown by the solid black lines.

In this study, we quantify the energy fluxes into the region associated with the remotely generated waves and eddies, and the convergence of this energy within the Indonesian Seas. We build on the work of the ref. 28 who estimate the eddy energy flux convergence from satellite altimetry. Their estimate assumes geostrophy, a balance between the sea surface height gradients and velocity, which is invalid near the equator. To extend their calculation to the equatorial region of the Indonesian Seas, we estimate the eddy energy fluxes directly using a regional ocean model. A snapshot of the SST from the model illustrates that the circulation of the Indonesian Seas is governed by complex topography with multiple internal seas, islands, narrow straits, and passages (Fig. 1). The snapshot captures the main features of the circulation, the ITF dominated by the western route (via Makassar Strait) as well as energetic mesoscale and submesoscale eddies, and the associated small-scale temperature filaments and fronts. Further details of the model configuration and energy flux calculation are given in the Methods.

## Results

### Eddy energy fluxes

**Fig. 2:**
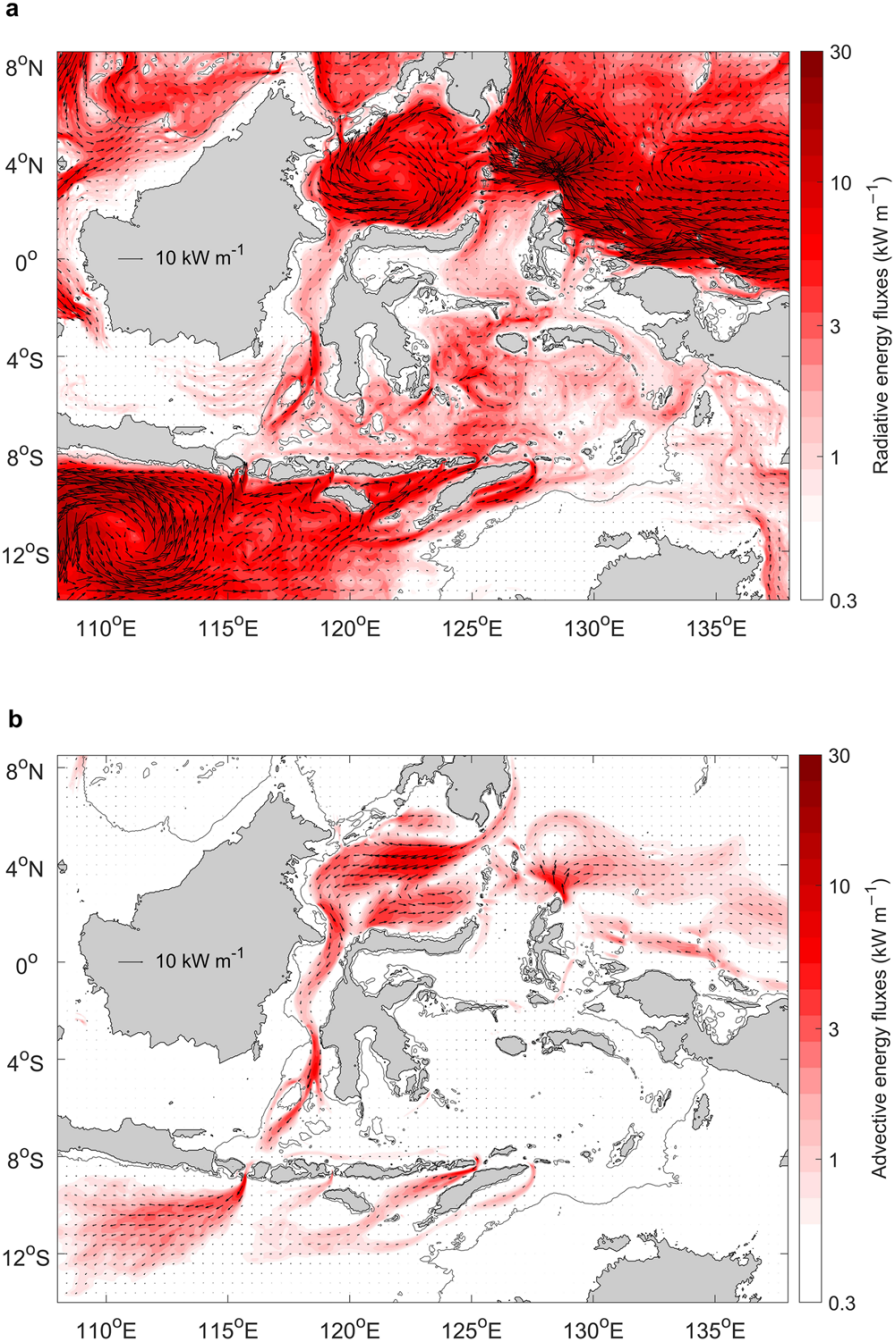
Eddy energy fluxes. Time-averaged, vertically-integrated (**a**) radiative and (**b**) advective eddy energy fluxes in (kW m^−1^; 1 kW m^−1^ = 10^3^ W m^−1^). The magnitude of the energy fluxes is shown in color on a logarithmic scale. Energy flux vectors represent 0.4° × 0.4° bin-averaged values. 200 m isobath is shown by the thin gray contour.

In the Western Pacific, the model-derived eddy energy fluxes are strong, with values ranging from 10–30 kW m^−1^ when time-averaged and vertically-integrated (Fig. 2). Similar to mid-latitudes, eddy energy fluxes in this region are westward and result from the westward propagation of eddies and Rossby waves in the equatorial Pacific Ocean^28^. Some of this energy is expected to lead to energy flux convergence (i.e., dissipation) near the western boundary of the Pacific Ocean^28^. In our model, a fraction of this eddy energy enters the Indonesian Seas through the entrance straits near the Maluku and Halmahera Seas with fluxes in the range of 3–10 kW m^−1^. Eddy energy also enters the Indonesian Seas further west, through the entrance straits near the Sulawesi Sea, where fluxes across the Sangihe Island chain can be up to 10 kW m^−1^. Inside the Indonesian Seas, the eddy energy from the Pacific Ocean continues to propagate south through Makassar Strait, the Maluku Sea, and the Halmahera Sea, but the magnitude of the energy fluxes decays rapidly in the interior.

In the northeastern Indian Ocean, the net (i.e., averaged over an eddy scale) eddy energy fluxes are also westward and of about 10 kW m^−1^. These energy fluxes are associated with the westward propagation of eddies generated from the instability of the ITF as it exits into the Indian Ocean. However, stronger eddy energy fluxes are also directed from the Indian Ocean towards the Indonesian Seas with magnitudes of 5–20 kW m^−1^ in the Lombok, Sape and Ombai Straits, and in Timor Passage. The time evolution of temperature and velocity anomalies in this region^29^ suggests that the eastward energy flux is associated with Kelvin waves generated in the equatorial Indian Ocean during the Monsoon Transition months. Kelvin waves propagate eastward along the Nusa Tenggara island chain and enter the Indonesian Seas, where they have been shown to drive strong variability on seasonal and intra-seasonal time scales^27,29^. The magnitude of the fluxes into the Indonesian Seas inferred here, O(10 kW m^−1^), is comparable to the magnitude of the internal tide energy fluxes estimated from tidal models^2,22^.

To compute the total eddy energy supply into the Indonesian Seas, we integrate the eddy energy fluxes across all straits and passages around the perimeter of the region (Table 1). We find that the total eddy energy flux into the region is 1.7 GW, with 37% coming from the Pacific Ocean and 63% from the Indian Ocean. Individual straits contribute between 0.1 to 0.4 GW to the energy flux into the region. The energy fluxes through all straits are directed towards the interior of the Indonesian Seas, and thus the Indonesian Seas are a net sink for eddy energy generated in the Indian and Pacific Oceans. This previously unaccounted remote energy source contributes energy to power the elevated mixing observed in the region.

**Table 1:** Eddy energy fluxes integrated across major straits and passages

	Radiative energy flux (GW)	Advective energy flux (GW)
Lombok Strait	0.36	−0.08
Sape Strait	0.15	−0.03
Ombai Strait	0.31	−0.11
Timor Passage	0.22	−0.07
Indian Ocean total	1.09	−0.34
Sulawesi Sea	−0.34	−0.02
Maluku Sea	−0.19	−0.04
Halmanhera Sea	−0.12	−0.02
Pacific Ocean total	−0.65	−0.08
Makassar Strait	−0.13	−0.23

Positive (negative) fluxes correspond to eastward/northward (westward/southward) flux direction.

In addition to the radiation of the eddy energy into the region, eddy energy can also be advected by the ocean currents. Consistent with the pathways of the ITF, eddy kinetic energy is also advected into the Indonesian Seas, primarily through the straits near the Sulawesi Sea on the Pacific Ocean side and advected out of the region through Lombok Strait, Ombai Strait, and Timor Passage on the Indian Ocean side (Fig. 2). We find that the total advective flux entering the Indonesian Seas from the Pacific Ocean is 0.1 GW and exiting to the Indian Ocean is 0.3 GW (Table 1). A significant amount of the eddy kinetic energy is generated locally within the Sulawesi Sea. This energy is advected southward by the ITF, corresponding to 0.2 GW of the advective energy flux in the Makassar Strait. The results suggest that the advection of the eddy kinetic energy makes a minor contribution to the total energy flux into the region.

### Eddy energy convergence and dissipation

**Fig. 3:**
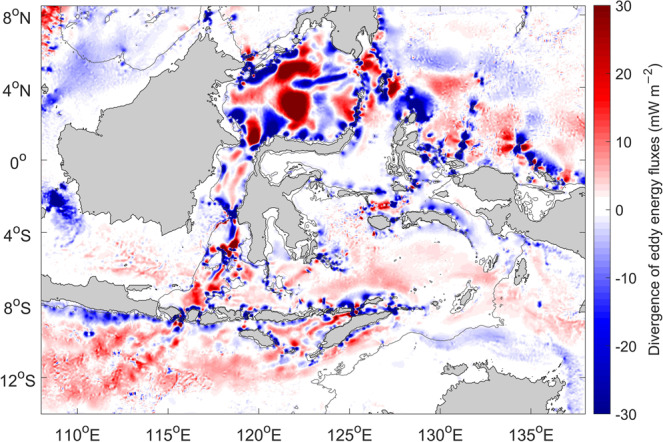
Divergence of time-averaged, vertically-integrated eddy energy fluxes in (mW m^−2^; 1 mW m^−2^ = 10^−3^ W m^−2^). Red and blue correspond to energy sources (positive) and sinks (negative). The color scale is saturated to show regions of relatively weak eddy energy sources in the interior. 200 m isobath is shown by the thin gray contour.

The spatial distribution of divergence (convergence) of the eddy energy flux indicates local sources (sinks) of the eddy energy (Fig. 3). Broad areas of divergence (positive, i.e., a source) occur mainly in the interior of the Sulawesi Sea and the Banda Sea. This eddy energy is likely generated in the interior of the internal seas through the instabilities of the ITF and wind-ocean interaction. Similarly, there is a broad area of divergence in the northeastern Indian Ocean associated with the instabilities of the ITF in the Indian Ocean. On the other hand, the eddy energy flux convergence (negative, i.e., a sink) is concentrated in narrow regions along topography and within the straits, suggesting that the eddy energy is likely dissipated through eddy (wave)-topography interaction. Idealized model simulations for western boundary regions of ocean basin^30^ show that significant eddy energy dissipation occurs at the western boundary regardless of whether the model topography is smooth or rough, with rough topography leading to enhanced energy dissipation. The magnitude of the eddy energy flux convergence is 10–30 mW m^−2^ along topography and reaches 100 mW m^−2^ within the straits. The distribution and magnitude of the eddy energy flux convergence, implying local energy dissipation, is in agreement with the turbulent energy dissipation observations^20,21^. Similar energy dissipation magnitudes and distribution were found for tidal energy dissipation^21,31^.

### Vertical distribution of eddy energy fluxes

**Fig. 4:**
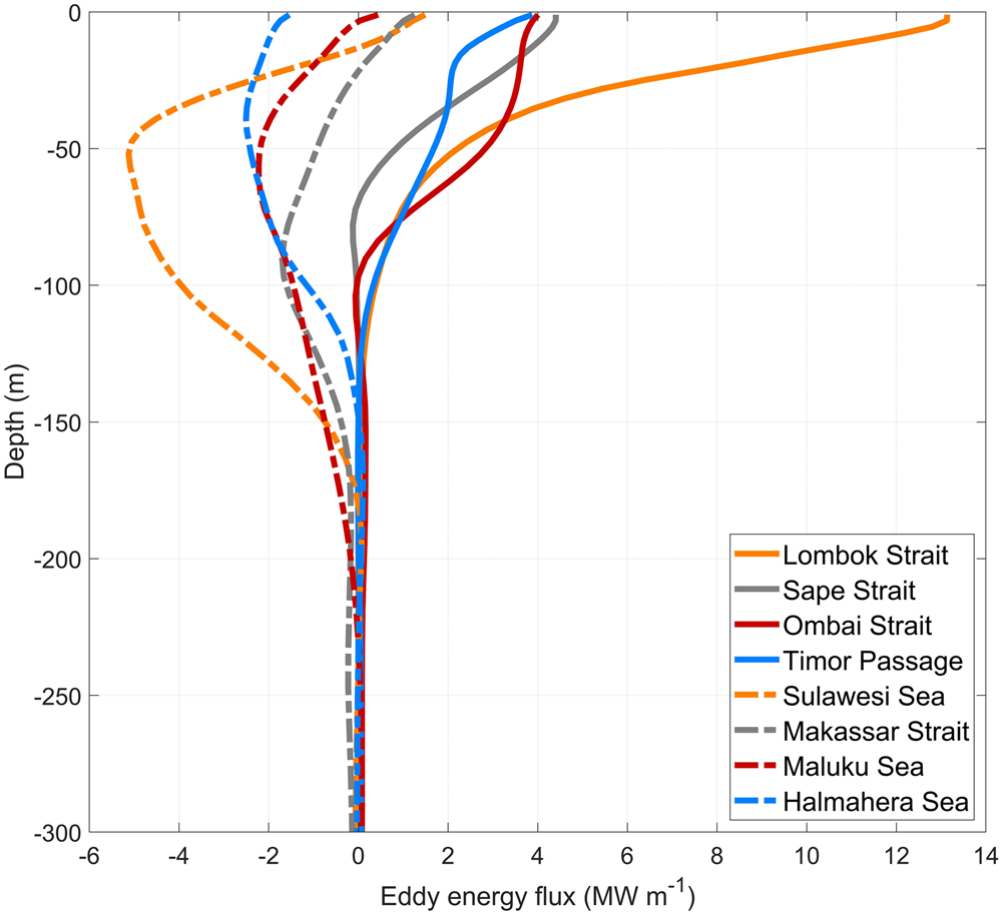
Vertical profiles of time-averaged, horizontally-integrated eddy energy fluxes in (MW m^−1^; 1 MW m^−1^ = 10^6^ W m^−1^) across major straits and passages. Fluxes on the Indian Ocean side are shown by solid lines and fluxes on the Pacific Ocean side are shown by dashed lines.

The propagating planetary (Rossby and Kelvin) waves and ocean eddies are generated by winds or instabilities of ocean currents and hence are surface intensified. Consistently, the vertical distribution of the energy fluxes integrated across major straits (Fig. 4) shows an enhancement in the upper 100–200 m of the ocean in all of the straits. On the Indian Ocean side, all fluxes are positive (i.e., into the Indonesian Seas) and are enhanced in the upper 50–100 m. Lombok Strait is characterized by the strongest eddy energy flux near the surface. The energy flux decreases from west to east in the Sape and Ombai Straits, and Timor Passage, consistent with the eastward propagation of Kelvin waves generated in the equatorial Indian Ocean. On the Pacific Ocean side, eddy fluxes are negative (i.e. also into the Indonesian Seas) and are characterized by a subsurface maximum at 50–100 m depth. They also extend deeper, to 200 m depth, in the water column. The difference in the vertical structure of energy fluxes is likely related to the difference in the thermocline depth between the Indian and Pacific oceans and its impact on wave propagation^27^.

## Discussion

Our results show that the Indonesian Seas are a sink of up to 1.7 GW of the remotely generated eddy energy flux. The eddy energy propagates into the Indonesian Seas through all the major straits along the Indian and western Pacific Ocean boundaries. The convergence of the eddy energy flux (i.e., energy dissipation) is enhanced in narrow regions along steep topography and within the straits with values of 10–100 mW m^−2^. Dissipated in those regions, the eddy energy flux can power turbulent energy dissipation and vertical mixing at rates comparable to tides and consistent with the recent turbulent energy dissipation observations in the Indonesian Seas^21^. Estimating eddy energy fluxes in ocean basins, ref. 28 finds that the total eddy energy convergence at the western boundaries of the North Pacific, South Pacific, and North Indian oceans is about 10–30 GW. Scaling up the area of the Indonesian Seas region to roughly match the size of the North Pacific Ocean, one obtains a comparable equivalent energy convergence, implying that the planetary waves and eddies are expected to be as important in the Indonesian Seas as they are near the western boundaries of ocean basins^28,32^.

Our results demonstrate a previously unaccounted energy source to sustain mixing in the upper ocean of the Indonesian Seas. This energy source is in addition to tidal energy more commonly identified in this region. In the absence of mixing processes powered remotely by winds, the mixing in the Indonesian Seas and hence its climate impacts would be misrepresented in global climate models. The remote energy generation and its propagation into the Indonesian Seas also imply a direct link between the wind forcing variability in the equatorial Indian and Pacific oceans and the SST and water-mass transformation within the Indonesian Seas, and hence a potential mechanism for climate teleconnections.

## Methods

### Model configuration

A high-resolution regional model of the Indonesian Seas is used in this study. The regional model is based on the Massachusetts Institute of Technology general circulation model (MITgcm)^33^. It is configured with 1/25° horizontal resolution and 100 vertical levels. The vertical resolution is 2.1 m at the surface, stretching to 263 m at depth. At the boundaries of the region, the model is forced by monthly-mean temperature, salinity, and velocity fields from a global eddy-resolving (0.1° resolution) ocean model ACCESS-OM2-01 forced by JRA55 do v1.3 (http://cosima.org.au)^34^. At the surface, the model is forced by the ACCESS-OM2-01 wind stress and also restored to the ACCESS-OM2-01 surface temperature and salinity fields with a 3-day restoring time scale. The outputs of the ACCESS-OM2-01 run forced by a repeat-year forcing^34^ are used to drive the regional model. Simulations are carried out with a K-profile parameterization (KPP) and Smagorinsky horizontal viscosity parameterization for sub-grid scale processes. The model bathymetry is from SRTM30_PLUS (https://topex.ucsd.edu). Our estimates are based on the last three years of the high-resolution regional model outputs. The regional model agrees well with observations^35^. Also, due to its relatively small domain size, the ocean state in the regional model stays consistent with that in the ACCESS-OM2-01 throughout the simulation. The validation of ACCESS-OM2-01, including in the Indonesian Seas region, is described in ref. 34.

### Eddy energy budget and fluxes

The total eddy kinetic energy budget^36^ can be written as

1 $$\frac{\partial \overline{E}}{\partial t}=-\nabla \cdot (\overline{{{{{{{{\boldsymbol{u}}}}}}}}E})-\nabla \cdot ({\rho }_{0}\overline{{{{{{{{\boldsymbol{u}}}}}}}}^{\prime} p^{\prime} })+\overline{b^{\prime} w^{\prime} }-{\rho }_{0}[\overline{u^{\prime} {{{{{{{\boldsymbol{u}}}}}}}}^{\prime} }\cdot \nabla \overline{u}+\overline{v^{\prime} {{{{{{{\boldsymbol{u}}}}}}}}^{\prime} }\cdot \nabla \overline{v}]+\overline{W}+\overline{\varepsilon },$$

where ***u*** = (*u*, *v*, *w*) is the total velocity, ${{{\overline{{{{{\boldsymbol{u}}}}}}}}}$ is the time-mean (3-year mean in our study) velocity, ${{{{{{{\boldsymbol{u}}}}}}}}^{\prime}$ is the eddy (i.e., time-varying) velocity; $p^{\prime}$ is the eddy pressure; $b^{\prime}$ is the eddy buoyancy; *ρ*_0_ is a reference density; and

2 $$\overline{E}=\frac{1}{2}{\rho }_{0}\big(\overline{{u^{\prime} }^{2}+{v^{\prime} }^{2}}\big)$$

is the eddy kinetic energy (EKE). Terms on the right-hand-side of the energy budget from left to right are the advection of EKE by total flow; the divergence of the radiative eddy energy flux; the energy exchange with potential energy (i.e., EKE production from baroclinic instability); the eddy-mean flow interaction (i.e., EKE production from barotropic instability); $\overline{W}$ is the work done by wind stress; and $\overline{\varepsilon }$ is the eddy energy dissipation.

Following ref. 28, we assume a steady-state and estimate the eddy radiative energy flux, ${\rho }_{0}\overline{{{{{{{{\boldsymbol{u}}}}}}}}^{\prime} p^{\prime} }$, and the eddy advective energy flux, ${\rho }_{0}\overline{{{{{{{{\boldsymbol{u}}}}}}}}E}$, and their divergence. Relating the eddy energy flux divergence to the energy dissipation, we neglect the contribution from potential energy (baroclinic instability), the eddy-mean flow interaction (barotropic instability), and the external forcing. These neglected terms are expected to be additional local sources of EKE within the region. Given that the focus here is on the remotely generated eddy energy and its propagation into the region (i.e., eddy fluxes), evaluating the contribution of the local sources of eddy energy due to flow instabilities is outside the scope of this study.

## Data Availability

The raw data that support figures are available at: https://github.com/ChengyuanPang/Remote-energy-sources. Full model outputs are available upon request to the corresponding author.

## Code availability

Code to reproduce key results is available at: https://github.com/ChengyuanPang/Remote-energy-sources.
